# Birth characteristics and risk of colorectal cancer: a study among Swedish twins

**DOI:** 10.1038/sj.bjc.6604918

**Published:** 2009-02-17

**Authors:** S Cnattingius, F Lundberg, A Iliadou

**Affiliations:** 1 Department of Medical Epidemiology and Biostatistics, Karolinska Institutet, PO Box 281, SE-171 77, Stockholm, Sweden; 2 Clinical Epidemiology Unit, Department of Medicine, Karolinska Institutet, Karolinska University Hospital, SE 171 76 Stockholm, Sweden

**Keywords:** birth length, birth weight, colorectal cancer, perinatal

## Abstract

Type-2 diabetes increases the risk of colorectal cancer, and is also associated with low birth weight. However, we found no evidence of associations between birth characteristics and risk of colorectal cancer (*m*=248) among Swedish twins.

Colorectal cancer is associated with type-2 diabetes (Hu *et al*, 1999) and with high concentrations of insulin-like growth factor I (IGF-I). Birth weight is commonly used as a proxy for foetal growth, and may be used as an indirect marker for foetal exposure to growth stimulating factors, such as IGFs (Osorio *et al*, 1996; Ong *et al*, 2000; Christou *et al*, 2001) and oestrogens (Petridou *et al*, 1990; Kaijser *et al*, 2000; Mucci *et al*, 2003). Low birth weight is associated with increased risk of type-2 diabetes (Harder *et al*, 2007), and high birth weight is associated with breast cancer (Silva Idos *et al*, 2008) and possibly also with prostate cancer (Tibblin *et al*, 1995; Platz *et al*, 1998; Ekbom *et al*, 1996, 2000; Boland *et al*, 2003; Nilsen *et al*, 2005b).

Little is known about early risk factors for colorectal cancer. Earlier studies of birth characteristics and colorectal cancer have been inconsistent (Sandhu *et al*, 2002; Nilsen *et al*, 2005a). We examined associations between birth characteristics and risk of colorectal cancer in like-sexed twins with known zygosity. The twin design also enabled us to take into account genetic and shared environmental factors in early life.

## Methods

In 1973, all like-sexed twins born in 1926–1958 included in the Swedish Twin Registry were sent a questionnaire, including questions of degree of likeness, anthropometric measures and lifestyle factors, with 81% response rate (Cederlof *et al*, 1961; Crumpacker *et al*, 1979; Lichtenstein *et al*, 2002). In this study, we restricted the cohort to twins with known zygosity, as determined by questions on childhood resemblance, which had earlier been validated with DNA markers (Lichtenstein *et al*, 2002). The person-unique national registration number, assigned to all Swedish citizens, permitted linkage among the Swedish Twin Registry, the Cancer and the Cause of Death Registers, and also enabled us to retrieve information from birth records. The study was approved by the research ethics committee of Karolinska Institutet.

Colorectal cancer cases were retrieved from the population-based Swedish Cancer Register, identified using the International Classification of Diseases (ICD) (ICD-7, ICD-8 and ICD-9 codes 153 and 154, and ICD-10 codes C18–C21).

Information on birth characteristics was abstracted from the original birth records. Correct birth identification of each twin within a pair was ensured by restricting the data collection to twin pairs who were both baptised and named at birth, or who reported birth order with mutual within-pair agreement in a telephonic interview conducted in 1998–2002 (Bergvall *et al*, 2007). Information from birth records included anthropometric measures at birth, gestational age, maternal age, parity and occupational status of both parents. Gestational age was based on the date of the first day of the last menstrual period. Socio-economic status (SES) at birth was based on information of parental occupation, and was classified according to the recommendations by Statistics Sweden (Swedish Socioeconomic Classification, 1983). Information on education and SES in adulthood was based on Swedish Census data of 1970 and 1980, respectively. Information on adult weight, height, smoking and alcohol consumption was collected through the 1973 questionnaire. Body mass index (BMI) was calculated as the ratio between the weight and squared height (kg m^2^). Alcohol consumption (estimated mean grams of alcohol per day) was classified according to the recommendations by World Health Organisation as low, medium or high (Ezzati, 2004). Other variables were categorised according to Table 1.

For this study, all like-sexed male and female twins with known zygosity born in 1926–1958 were considered. For the 32 011 twins who were alive and without earlier diagnosis of colorectal cancer at start of follow-up in 1973, the birth record coverage was 73%. Restrictions were because of missing birth weight data in birth records (*n*=6293) and uncertain correct identification of each twin within a twin pair at birth (*n*=2381), resulting in a final study population of 23 337 twins (including 11 419 male and 11 918 female twins).

### Statistical analyses

In the cohort analysis, risk time (person–years) was accrued from the time of entry (1 January, 1973) until a first diagnosis of colorectal cancer or censored at the date of first emigration from Sweden, death or end of follow-up (31 December 2006). Cox proportional hazard models were used to estimate hazard ratios (HRs) for colorectal cancer with age (measured in months) as the underlying time scale, and robust standard error estimates to account for the dependence within twin pairs.

We estimated the independent risk of colorectal cancer for all exposure variables (Table 1). Different adjusted models were used in the cohort analyses to estimate risk (Table 2, models 1–4). All twins share early environmental factors, dizygotic and monozygotic twins share 50 and 100% of their segregating genes, respectively. To account for familial (genetic and shared early environmental) factors, we calculated mean (±s.d.) birth weight and birth length among dizygotic and monozygotic twin pairs, discordant for colorectal cancer. Analyses were carried out in PROC PHREG in SAS 9.2.

## Results

In our cohort of 23 337 twins, 248 developed colorectal cancer during the follow-up period. Table 1 shows the distribution of birth and adult characteristics and crude HRs for colorectal cancer. Compared with males, females had a reduced risk. Risk increased with birth weight, but no risk estimates were significant. Other birth characteristics and parental factors at delivery did not influence the risk. Overweight in adulthood (BMI ⩾25.0) was associated with an increased risk of colorectal cancer.

Table 2 shows birth weight and birth length categories and HRs. Generally, there were no significant associations between birth weight or birth length and risk in the (crude or adjusted) models. When we also adjusted in a subsample for adult characteristics, a long birth length (⩾50 cm) appeared to be protective (HR 0.56; 95% CI 0.33–0.94). However, this was probably explained by a selection effect: a reduction in risk was also obtained when we included the same number of individuals (*n*=8757) in the model and only adjusted for birth characteristics (HR 0.57; 95% CI 0.34–0.94). There were no significant interactions between sex and birth weight or birth length with respect to risk, in neither the crude (*P*=0.29 and *P*=0.51, respectively) nor the fully adjusted models (*P*=0.51 and *P*=0.69, respectively).

To account for familial (genetic or shared environmental) factors, we also carried out analyses within disease-discordant twin pairs. In dizygotic disease-discordant twin pairs (*n*=144), the mean birth weight (s.d.) was 2761 g (507) and 2756 g (487) among cases and co-twin controls, respectively (*P*=0.94). In monozygotic disease-discordant twin pairs (*n*=86), the corresponding mean birth weights (s.d.) were 2610 g (495) and 2642 g (550), respectively (*P*=0.69). Similarly, there were no differences in mean birth length within dizygotic or monozygotic disease-discordant twin pairs (data not shown).

## Discussion

In a cohort study of Swedish twins, we found no evidence that birth characteristics influenced the risk of colorectal cancer. There were also no differences in mean birth weight and birth length among disease-discordant dizygotic and monozygotic twin pairs. As analyses within disease-discordant twin pairs account for familial (genetic and early environmental) factors and perfectly match for gestational age, these negative findings further strengthen the hypothesis that foetal growth is not related to offspring risk of colorectal cancer.

A J-shaped relation between self-reported birth weight and colorectal cancer was initially reported by a British study, including 96 cases (Sandhu *et al*, 2002). A Norwegian study, including 247 cases, found that the risk was increased among males with a short birth length (Nilsen *et al*, 2005a). However, the sex-specific analyses included only 150 male and 97 female cancer cases, and interaction analyses between birth characteristics and sex with respect to risk of colorectal cancer were not reported. In our negative study, we found no significant interactions between sex and birth characteristics with respect to risk of colorectal cancer.

The generalisability of results from twin studies may be questionable as twins are in general more growth restricted than singletons, have shorter gestational age, and because they may differ in prenatal environment and upbringing. However, the incidence of colorectal cancer does not appear to be different in twins compared with singletons (Verkasalo *et al*, 1999). As studies within twin pairs adjust for gestational age, differences in birth weight within twin pairs reflect the differences in foetal growth, and genetic factors are fully taken into account when analyses are restricted to monozygotic twin pairs. Thus, for the research question of the effect of foetal growth on offspring risk of disease, twin studies have a high internal validity.

This study lends no support for an association between birth characteristics and risk of colorectal cancer. The conflicting results from the few earlier studies may be because of limited statistical power, but it is relevant that the underlying biological reasons for an association with birth characteristics remain speculative.

## Figures and Tables

**Table 1:** Numbers and hazard ratios^a^ (HRs) with 95% confidence interval (CI) of colorectal cancer as a function of maternal, birth and adult characteristics among like-sexed Swedish twins born in 1926–1958

	**Study population**		**Colorectal cancer**		
	***n***	***n***	**(%)**	**HR^a^**	**95% CI**
Total	23 337	248	1.1		
					
*Zygosity*					
Monozygous	9141	95	1.0	1.00	
Dizygous	14 196	153	1.1	0.99	(0.76–1.28)
					
*Sex*					
Male	11 419	146	1.3	1.00	
Female	11 918	102	0.9	0.63	(0.49–0.81)
					
*Birth year*					
1926–1936	4282	128	3.0	1.00	
1937–1943	5092	62	1.2	0.85	(0.60–1.21)
1944–1950	7029	35	0.5	0.71	(0.44–1.13)
1951–1958	6934	23	0.3	1.28	(0.72–2.26)
					
*Birth weight, g*					
<2500	8904	86	1.0	0.92	(0.68–1.25)
2500–2999	8444	86	1.0	1.00	
3000–	5989	76	1.3	1.21	(0.89–1.65)
					
*Birth length, cm*					
<47	7220	68	0.9	0.84	(0.59–1.20)
47–49	6761	79	1.2	1.00	
⩾50	9183	101	1.1	0.97	(0.74–1.28)
Missing	173	0	0.0		
					
*Gestational age, weeks*					
32–34	3009	22	0.7	0.73	(0.47–1.14)
35–36	4654	49	1.1	0.95	(0.69–1.32)
37–41	13 761	149	1.1	1.00	
42–45	816	9	1.1	1.02	(0.52–1.98)
Missing	1097	19	1.7		
					
*Mother's age, years*					
⩽19	629	7	1.1	1.21	(0.56–2.62)
20–24	3926	44	1.1	1.12	(0.76–1.65)
25–29	6966	71	1.0	1.00	
30–34	6434	66	1.0	0.98	(0.70–1.38)
⩾35	5368	60	1.1	0.98	(0.70–1.39)
Missing	14	0	0.0		
					
*Maternal parity*					
Primipara	7230	66	0.9	1.00	
Multipara	15 799	175	1.1	1.25	(0.93–1.67)
Missing	308	7	2.3		
					
*Parental SES at birth*					
Blue-collar worker	10 100	98	1.0	1.33	(0.86–2.06)
White-collar worker	4483	26	0.6	1.00	
Self-employed	2877	19	0.7	0.94	(0.52–1.70)
Missing	5877	105	1.8		
					
*SES in adulthood*					
Blue-collar worker	9101	96	1.1	0.95	(0.72–1.26)
White-collar worker	8657	101	1.2	1.00	
Self-employed	1418	18	1.3	0.86	(0.52–1.42)
Missing	4161	33	0.8		
					
*Education level*					
Compulsory	10 708	137	1.3	1.00	
More than compulsory	8618	95	1.1	1.01	(0.77–1.32)
Missing	4011	16	0.4		
					
*BMI in 1973, kg m* ^*−2*^					
<18.5	2242	14	0.6	1.36	(0.76–2.44)
18.5–24.9	15 792	142	0.9	1.00	
⩾25	2283	54	2.4	1.60	(1.15–2.23)
Missing	3020	38	1.3		
					
*Height in 1973 (quartiles)* b					
1st	4714	53	1.1	0.89	(0.62–1.28)
2nd	5096	58	1.1	1.00	
3rd	4464	40	0.9	0.82	(0.54–1.23)
4th	6093	59	1.0	1.00	(0.70–1.44)
Missing	2970	38	1.3		
					
*Smoking status in 1973*					
Never	8588	96	1.1	1.00	
Ever	11 047	108	1.0	0.97	(0.74–1.28)
Missing	3702	44	1.2		
					
*Alcohol consumption in 1973*					
None	6836	63	0.9	1.00	
Low	11 645	133	1.1	1.29	(0.95–1.74)
Moderately high	1197	11	0.9	1.01	(0.53–1.91)
Missing	3659	41	1.1		

BMI=body mass index; SES=socio-economic status.

a Hazard ratios are stratified for birth cohort and adjusted for age at end of follow-up.

b Sex-specific height quartiles.

**Table 2:** Unadjusted and adjusted hazard ratios (HRs) with 95% confidence intervals (CI) of colorectal cancer in relation to birth weight and birth length in the cohort analyses of Swedish like-sexed twins born in 1926–1958

	**Study population**	**Colorectal cancer**		**HR (95% CI), adjusted for**	
**Birth weight, g**	***N***	***n***	**%**	**Birth characteristics^a^ (*n*=22 240)**	**Birth and maternal characteristics^b^ (*n*=16 703)**
<2500	8904	86	1.0	1.01 (0.72–1.40)	1.07 (0.69–1.64)
2500–2999	8444	86	1.0	1.00	1.00
⩾3000	5989	76	1.3	1.15 (0.82–1.61)	1.04 (0.66–1.64)
					

**Birth length, cm**				**(*n*=22 097)**	**(*n*=16 649)**
<47	7220	68	0.9	0.94 (0.65–1.34)	0.92 (0.58–1.47)
47–49	6761	79	1.0	1.00	1.00
⩾50	9183	101	1.1	0.88 (0.64–1.20)	0.72 (0.48–1.09)

All analyses are stratified by birth year and have accounted for the clustered data structure and between-cluster effect.

a Adjusted for zygosity, sex and gestational age.

b Adjusted for birth characteristics, mother's age, parity and socioeconomic status at birth.
